# CMNet: an asymmetric dual-branch network for accurate cotton segmentation

**DOI:** 10.3389/fpls.2025.1692647

**Published:** 2026-03-03

**Authors:** Gengrong Zhang, Halidanmu Abudukelimu, Mayilamu Musideke, Shuqin Wu, Abudukelimu Abulizi, Cuiqin Guo, Yajun Zhang

**Affiliations:** 1 School of Information Management, Xinjiang University of Finance and Economics, Urumqi, Xinjiang, China; 2 School of Software, Xinjiang University, Urumqi, Xinjiang, China

**Keywords:** dual-branch, cotton segmentation, SS2D, deformable convolution, scSE

## Abstract

In agricultural automation, precise cotton segmentation is a key step for tasks such as intelligent harvesting and yield estimation. However, in complex field environments, factors such as background interference and irregular target shapes severely affect segmentation accuracy. Existing deep learning methods offer certain advantages but still generally suffer from limitations including insufficient accuracy, over-segmentation, and misidentification. To address these challenges, this study proposes a novel dual-branch cotton segmentation network, Cotton-aware Mamba-enhanced UNet (CMNet), which optimizes the ParaTransCNN architecture by incorporating the 2D Selective Scan (SS2D) module to replace the original Transformer branch, effectively balancing the extraction of local details and global semantic information while reducing computational burden. To enhance the model’s perception of irregularly shaped cotton, a Deformable Convolutional Networks v1 (DCNv1) module is integrated into the Vision Mamba (VMamba) branch, further improving the delineation of target boundaries. Additionally, an Atrous Spatial Pyramid Pooling (ASPP) module is introduced at the end of the Convolutional Neural Network (CNN) branch to strengthen multi-scale feature representation. To optimize the fusion of channel and spatial information, the Spatial and Channel Squeeze-and-Excitation (scSE) attention mechanism replaces the original module, enhancing feature modeling capability. Experimental results on an in-field cotton image dataset demonstrate that CMNet outperforms existing mainstream methods, achieving Dice, mIoU, and Accuracy of 91.06%, 84.18%, and 98.10%, respectively, while reducing parameter count and computational complexity, thus exhibiting excellent performance. Furthermore, generalization experiments on multiple other plant datasets also achieved outstanding results, validating the model’s adaptability and potential for broader applications in multi-crop segmentation tasks, providing valuable insights for smart agriculture segmentation research. The source code and dataset of this work are publicly available at https://github.com/halidanmu/CMNet.git.

## 1 Introduction

Cotton, as one of the world’s important cash crops, is widely used in textile, medicine, and chemical industries, among others (Tlatlaa et al., 2023). With the continuous expansion of cultivation scale, how to efficiently manage cotton growth, monitor plant health, and reduce resource waste has become a major challenge facing the current cotton industry. Among these challenges, pest infestations (Carneiro et al., 2025), climate change (Zhu et al., 2023), and low harvesting efficiency (Singh et al., 2024) are key factors affecting cotton production. In particular, low harvesting efficiency stands out as the most critical issue, as it directly impacts both the yield and quality of cotton. Existing cotton harvesting methods mainly include hand picking, mechanized harvesting, and semi-mechanized harvesting. Traditional cotton harvesting primarily relies on hand picking, which ensures high-quality harvesting but often fails to meet the demands of large-scale production due to limitations in labor cost and labor efficiency, and has thus gradually exited the mainstream (Gharakhani et al., 2024). Mechanized harvesting significantly improves harvesting efficiency and reduces labor cost but its drawbacks are more pronounced. During cotton harvesting, fibers are prone to mixing with impurities, reducing quality and textile value. Moreover, mechanical harvesters require uniform field conditions and optimal cotton maturity, and improper operation may affect subsequent planting. In addition, the high cost of equipment poses a burden for smallholders and economically underdeveloped regions. Although semi-mechanized harvesting improves picking efficiency to some extent, its performance remains unstable. In addition, the complex environment of cotton fields and the uneven distribution of cotton plants can easily interfere with picking accuracy and operational efficiency. Therefore, achieving intelligent and refined management of the cotton harvesting process has become an urgent issue to be addressed. Artificial intelligence (AI)-based visual perception technologies offer new approaches to this goal, among which high-precision cotton structure segmentation serves as a fundamental basis for intelligent recognition and automated harvesting.

With the rapid advancement of AI, computer vision technology has been increasingly applied in fields such as medical image analysis (Wang et al., 2025; Chen et al., 2025), autonomous driving (Luo et al., 2025), and urban planning (Hu et al., 2025). As one of the core approaches, image segmentation divides an image into multiple regions or objects with specific semantic or structural meanings, effectively distinguishing complex backgrounds from target objects, and serves as a crucial step from image processing to image analysis. In recent years, image segmentation has been increasingly applied in agriculture, assisting growers in accurately assessing crop health, detecting pest- and disease-affected areas, and distinguishing between different crop types, thereby providing real-time data support and decision-making guidance for agricultural production (Zhang and Mu, 2024; Guo et al., 2025; Dong et al., 2025).

However, various challenges still hinder existing segmentation models from achieving high accuracy in cotton field scenarios. Firstly, the field environment is complex, and images collected are easily affected by variations in lighting, weather, and background, which reduces segmentation accuracy (Rodriguez-Sanchez et al., 2022). Secondly, the irregular spherical shape of cotton and the fuzz on its surface cause blurred edges, while the irregularity of its shape and distribution further increases the segmentation difficulty. Meanwhile, the uneven spatial distribution of cotton leads to adhesion in dense regions and missed detections of small targets in sparse regions due to feature loss. To address these challenges, several studies have attempted to employ various segmentation models to improve performance. Tan et al. (2025) combined an improved DM-Count with a Segment Anything Model (SAM) to achieve high-precision predictions of cotton boll counts, sizes, and lint yield from Unmanned Aerial Vehicle (UAV)-acquired images. However, this method exhibits poor robustness under low-light conditions and can misidentify visually similar objects (e.g., white flags) as cotton. Bolouri et al. (2024) developed CottonSense, a high-throughput phenotyping system based on Red-Green-Blue-Depth (RGB-D) cameras and an optimized Mask R-CNN model for edge computing. Using a tracking-based enumeration algorithm, the system enhances field-level statistical efficiency, providing low-cost agronomic support for high-yield breeding and crop management. However, the accuracy of this model remains relatively low.

**Figure 1:**
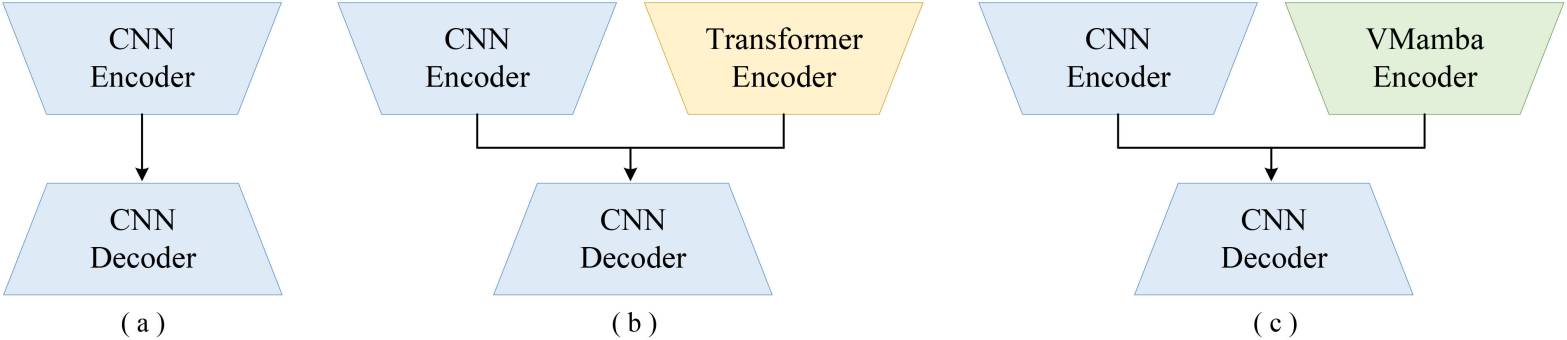
Architectures of three image segmentation models: **(a)** classic U-Net; **(b)** ParaTransCNN; **(c)** CMNet.

In summary, the irregular shape, diverse scales, and complex edge structures of cotton make existing methods still prone to significant interference and insufficient segmentation accuracy in complex field environments. The ParaTransCNN model (Sun et al., 2024) is a dual-branch segmentation network that integrates CNNs with Transformer structures. It fully leverages both global information and local details, and employs a Squeeze-and-Excitation (SE) module (Hu et al., 2018) for refined feature extraction, thereby producing high-quality segmentation results. As shown in **Figure 1**, this study proposes an improved cotton segmentation network—Cotton-aware Mamba-enhanced UNet (CMNet)—based on ParaTransCNN. The proposed network fully exploits the complementary characteristics of the dual-branch architecture and integrates multiple advanced modules to enhance segmentation accuracy and efficiency, enabling precise representation of cotton with complex morphology. The main contributions of this study are summarized as follows:

1. The 2D-Selective-Scan (SS2D) module (Liu et al., 2024b) replaces the Transformer module in the ParaTransCNN architecture to construct the Vision Mamba (VMamba) branch, enabling the acquisition of global semantic information while reducing computational cost. By introducing Deformable Convolutional Networks v1 (DCNv1) (Dai et al., 2017), this branch can adaptively learn the spatial positions of pixels to extract the polygonal features of cotton.
2. An Atrous Spatial Pyramid Pooling (ASPP) module (Chen et al., 2017) is integrated at the end of the CNN branch to capture multi-scale contextual information, enabling comprehensive extraction of cotton features.
3. The feature fusion module replaces the SE module with the Spatial and Channel Squeeze-and-Excitation (scSE) module (Roy et al., 2018), allowing the network to autonomously balance the optimization of spatial and channel features under different scenarios.

The remainder of this paper is organized as follows: Section 2 reviews the related work of this study; Section 3 describes the methods employed in this paper; Section 4 presents the experimental data and parameter settings; Section 5 analyzes the experimental results; Section 6 discusses the findings and limitations of this work; and Section 7 concludes the paper with a summary and future perspectives.

## 2 Related work

### 2.1 Image segmentation

With the rapid development of computer vision technology, significant technical advancements have been made in image segmentation, which is one of the core tasks of computer vision. Traditional segmentation methods were once the mainstream approaches for image segmentation. However, with the introduction of deep learning technology, models can automatically learn image features, achieving segmentation results with higher accuracy than traditional methods and demonstrating stronger robustness in complex scenarios.

#### 2.1.1 Traditional image segmentation techniques

Traditional image segmentation techniques primarily rely on manually designed features and mathematical criteria to establish low-level similarity relationships between pixels for target extraction. As one of the early methods, threshold segmentation divides an image into foreground and background by setting a gray-level threshold, performing well in images with simple structures and clear contrast (Otsu, 1975), but struggling to handle complex backgrounds or overlapping gray-level distributions. Edge-based segmentation methods detect abrupt changes in the grayscale gradient to locate object boundaries (Sobel and Feldman, 1968; Canny, 1986), yet they are prone to boundary discontinuities or incomplete closure under noisy or blurred conditions. To obtain more coherent and complete segmentation results, region-based methods enforce consistency within regions based on spatial similarity among pixels. However, their performance depends heavily on initial parameter settings and is susceptible to local optima. Subsequently, graph-based segmentation methods, such as the minimum cut (Min-Cut) approach (Boykov and Jolly, 2001), model the image as a weighted undirected graph and capture image structure from a global perspective, albeit at a high computational cost.

In summary, although traditional image segmentation methods remain somewhat practical in specific scenarios, they often perform poorly when dealing with complex backgrounds or variations in target scales. With the rise of deep learning, automatic methods that learn high-level semantic information for image segmentation have gradually superseded traditional approaches, becoming the mainstream focus of current research.

#### 2.1.2 AI-based image segmentation technology

With the rapid development of computer vision technology, some studies based on traditional machine learning algorithms are built upon manually designed low-level image features and achieve segmentation by integrating classifiers (Bhimavarapu et al., 2024). However, such methods are less capable of selecting features and generalizing models, making it difficult to adapt to complex backgrounds and unstructured scenes. To overcome the aforementioned limitations, researchers have begun leveraging CNN-based models, such as Fully Convolutional Networks (FCN) (Long et al., 2015) and U-Net architectures (Ronneberger et al., 2015), to automatically learn semantic features from images (Huang et al., 2019). Furthermore, researchers have introduced Transformer architectures into image segmentation tasks, demonstrating outstanding capabilities in global feature modeling. However, Transformer models generally have a large number of parameters and require high computational resources. The Mamba architecture (Gu and Dao, 2023) efficiently captures long-range dependencies through state-space modeling. As a variant of Mamba, VMamba maintains linear complexity while achieving long-range dependency modeling (Liu et al., 2024b), providing new insights for image segmentation (Ruan et al., 2024).

To address the issue of insufficient recognition accuracy of a single segmentation model in complex backgrounds, two-stage architectures decouple object detection from pixel-level segmentation tasks. Mask R-CNN (He et al., 2017) incorporates a fully convolutional branch based on the object detection of Faster R-CNN, achieving instance-level mask prediction and significantly improving segmentation accuracy for small objects and overlapping areas. With the development of transfer learning, the ability of pre-trained models to transfer knowledge across different tasks or data domains has been significantly enhanced, providing an effective approach to addressing challenges posed by complex backgrounds (Bao et al., 2024). For example, Wang and Li (2025) proposed an ultrasonic-based gesture recognition system for intelligent device unlocking, which leverages transfer learning to achieve user-specific real-time authentication while preserving privacy. Moreover, multi-modal image segmentation methods enhance model robustness to interferences such as illumination variations by integrating multi-source information, including RGB, thermal infrared, depth, or hyperspectral data, thereby providing abundant algorithmic support and technical solutions for the agricultural domain.

### 2.2 Advancements in cotton segmentation research

The goal of cotton image segmentation is to accurately extract mature cotton boll regions in complex field environments, providing critical data support for harvesting and thereby promoting efficient and sustainable agricultural production. Early cotton image segmentation methods were primarily based on traditional image processing techniques, utilizing low-level features such as color, shape, and texture to achieve target extraction (Wang et al., 2006; Wei et al., 2008). Although these methods are simple to implement, they exhibit poor robustness under complex conditions such as lighting variations or blurred details, making them difficult to adapt to real-world field environments. Subsequently, researchers introduced segmentation methods based on traditional machine learning, which, using algorithms such as the SM spectral clustering method (Liu et al., 2022), effectively enhance the accuracy and real-time performance of cotton image segmentation in complex environments. Although these methods exhibit better generalization than traditional image processing techniques, they still struggle to capture high-level semantic information in complex scenarios.

With the rise of deep learning technology, CNN-based image segmentation methods have been widely applied in the cotton domain. Singh et al. (2022) constructed pixel-level segmentation models for cotton and sky based on VGG16, InceptionV3, and ResNet34, respectively, and the results indicated that the InceptionV3 model achieved the best performance. To further improve segmentation accuracy, the same team alleviated the vanishing gradient problem through residual connections and utilized skip connections to achieve efficient feature fusion, thereby obtaining the highest IoU value (Singh et al., 2023). In addition, they optimized the Vision Transformer (ViT) model, achieving segmentation accuracy superior to that of CNN-based models (Singh et al., 2025). As the complexity of segmentation tasks continues to increase, relying solely on simple model architectures has become insufficient to capture the semantic information in cotton images. Studies have shown that introducing hierarchical structural networks and attention mechanisms can effectively extract multi-scale feature relationships, enhance the understanding of target spatial distributions, and capture semantic information in complex images (Yang et al., 2022; Yuan et al., 2025). Yan et al. (2023) innovatively combined CoAtNet and Xception as the backbone network for cotton organ segmentation, achieving outstanding performance in delineating cotton leaf edges. Moreover, Liu et al. (2024a) replaced the downsampling modules in the traditional U-Net with ResNet50 and incorporated Coordinate Attention (CA) and the HardSwish activation function, significantly improving the accuracy and robustness of cotton image segmentation. To enhance the operational efficiency of segmentation models, Xu et al. (2025) proposed an improved DeepLabV3+ model that adopts a lightweight two-stage segmentation architecture, effectively improving segmentation precision. Furthermore, Bolouri et al. (2024) developed the CottonSense high-throughput phenotyping system based on RGB-D cameras and an optimized Mask R-CNN model, achieving complete model deployment. The system demonstrated high accuracy and real-time performance under field conditions, providing strong data support for high-yield cotton breeding and agronomic management, though challenges in achieving precise segmentation still remain.

**Figure 2:**
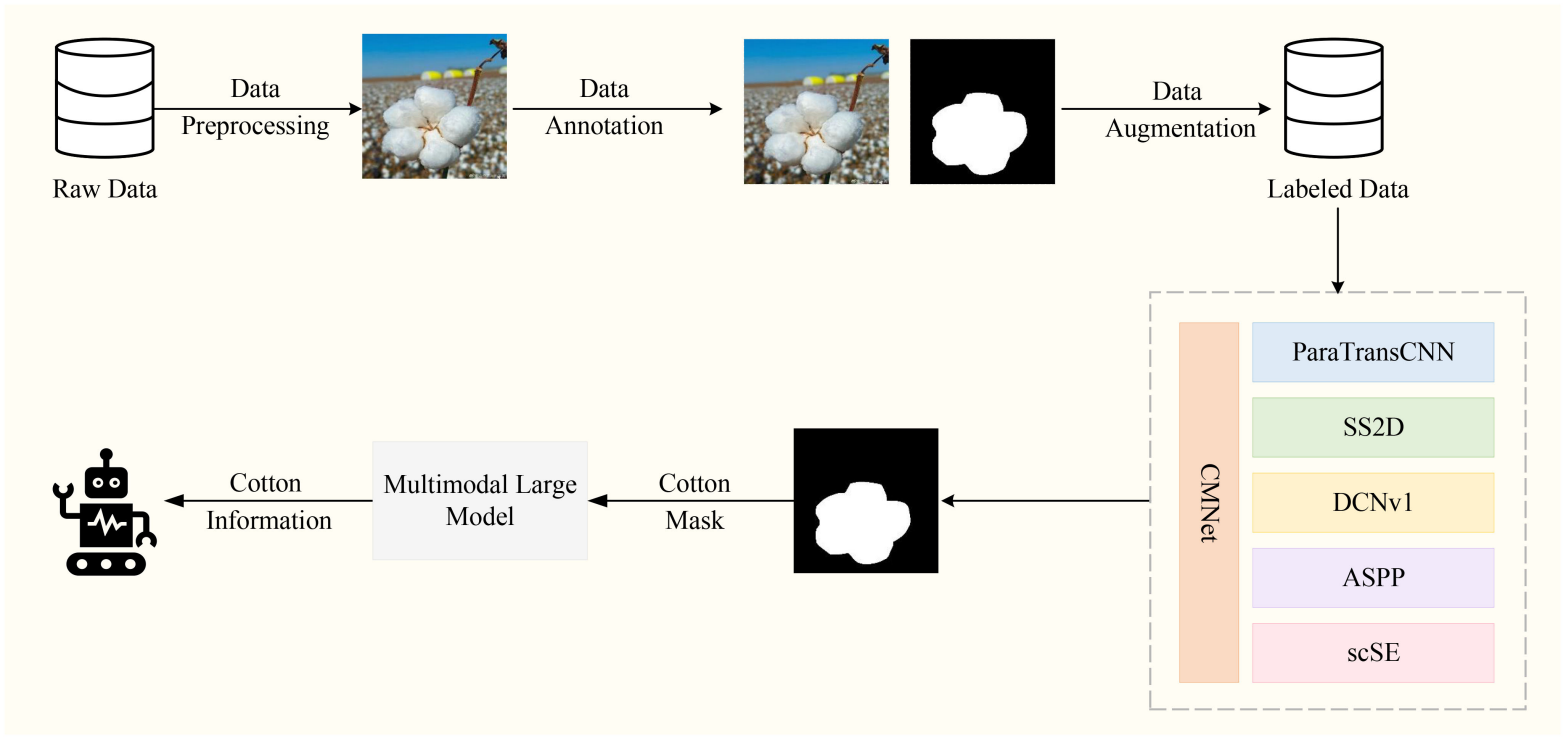
Workflow for this study.

Despite the remarkable progress in cotton segmentation research in recent years, several challenges remain to be addressed. Although some segmentation models have achieved improvements in accuracy and efficiency, their robustness under complex cotton growth environments is still insufficient (Tan et al., 2025; Bolouri et al., 2024). Therefore, as illustrated in **Figure 2**, this study proposes a comprehensive workflow: first, a high-quality dataset was constructed through data cleaning, augmentation, and expansion; second, the CMNet model is designed to tackle the recognition challenges posed by complex backgrounds and irregular cotton shapes; finally, the images identified by the model are fed into a fine-tuned Contrastive Language–Image Pretraining (CLIP) model to generate corresponding textual descriptions, facilitating automated cotton picking by robotic systems.

## 3 Methods

### 3.1 CMNet architecture

To address the key challenges in cotton segmentation tasks, this study proposes an improved dual- branch model based on ParaTransCNN (Sun et al., 2024). The ParaTransCNN model adopts a U-shaped encoder–decoder architecture, in which the two parallel encoder branches complement each other. Specifically, the Transformer branch divides the input image into small patches to extract global contextual information, while the CNN branch employs ResNet as its backbone to capture local details through successive downsampling operations. Meanwhile, an SE module is introduced between the two branches to enhance informative features and suppress irrelevant ones, thereby obtaining semantically rich fused representations. Finally, multi-scale features from each encoder stage are transmitted to the decoder, where fine-grained segmentation masks are generated.

**Figure 3:**
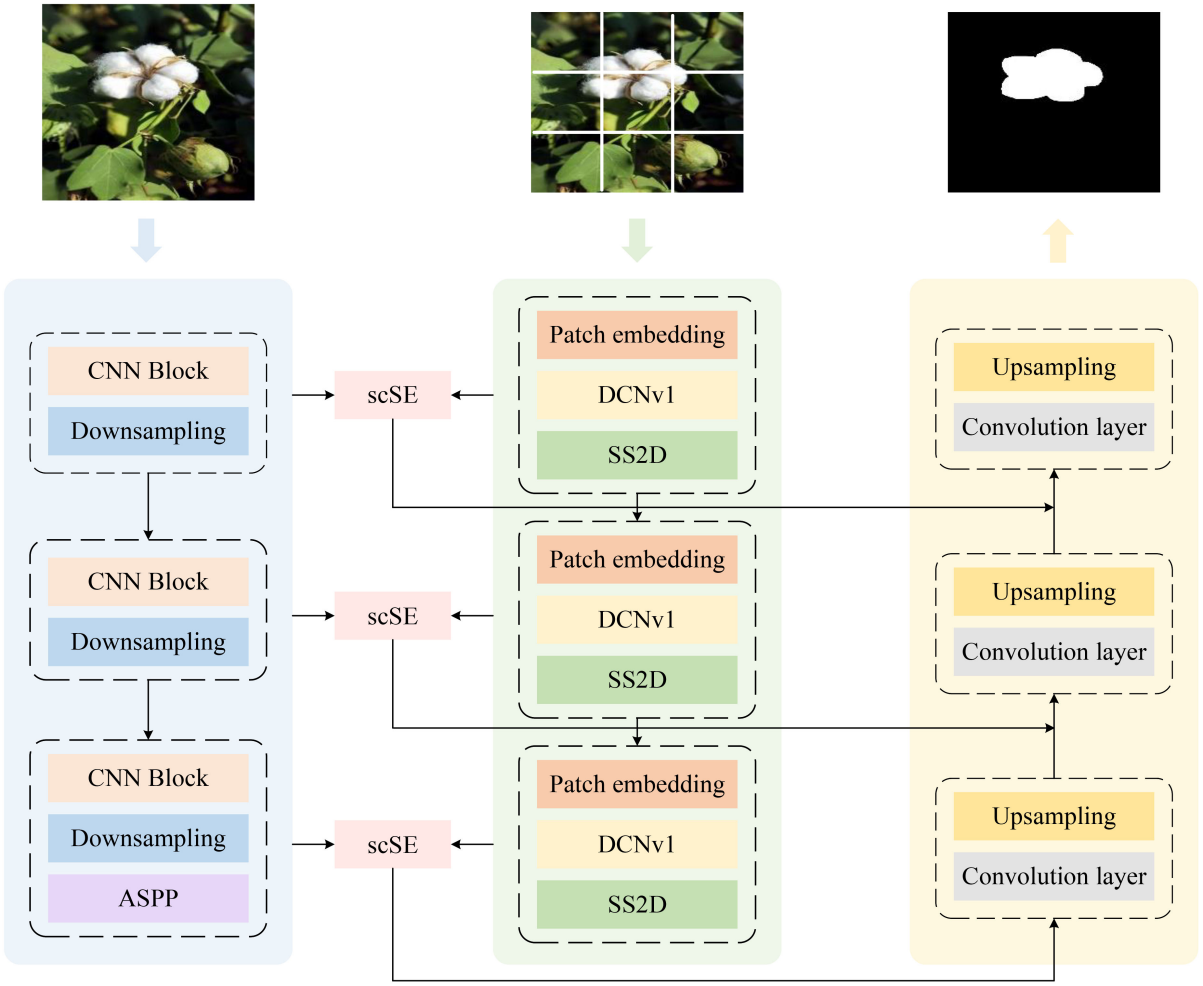
Architecture of CMNet.

However, in cotton harvesting scenarios, models often face segmentation challenges arising from complex environments and irregularly shaped targets. To address this issue, as illustrated in **Figure 3**, we propose a cotton segmentation model named CMNet. First, the cotton images are fed into the improved dual-branch encoder, where the Transformer branch incorporates the SS2D module to replace the conventional Transformer, thereby significantly reducing computational complexity while preserving global modeling capability. Meanwhile, the DCNv1 module is integrated into this branch to enhance feature learning for irregular cotton shapes. In the CNN branch, an ASPP module is added at the end to expand the receptive field and capture multi-scale contextual features, effectively enhancing the extraction of local textures and fine details. To achieve efficient interaction and fusion between the two branches, the SE module is replaced with an scSE module, which simultaneously focuses on channel and spatial dimensions to strengthen the representation of key features. Finally, the fused multi-level features are passed into the decoder, where a series of upsampling and convolution operations are performed to generate high-precision cotton segmentation masks.

### 3.2 SS2D module

Transformer relies on the Multi-Head Self-Attention (MHSA) mechanism, which models pairwise interactions among all global positions in each layer. Although this approach enhances the extraction of global semantic information, it often neglects the continuity of local structures, leading to performance limitations under low-contrast or blurred-boundary conditions, while also introducing substantial computational and memory overhead. In contrast, cotton segmentation tasks often involve complex backgrounds and irregular target boundaries, imposing higher demands on the model’s local structural perception ability and computational efficiency. The State Space Model (SSM) computes outputs by recursively updating hidden states in conjunction with the current inputs, offering higher computational efficiency and lower resource consumption compared to the Transformer architecture. Building upon SSM, the Mamba model (Gu and Dao, 2023) introduces the Selective State Space Mechanism, which dynamically selects the transmission paths of input features to achieve efficient long-range context modeling. Furthermore, VMamba (Liu et al., 2024b) extends the application of Mamba to the visual domain by integrating a scanning mechanism, thereby enhancing the model’s ability to capture spatial structures in images. This design is particularly suitable for image processing tasks with variable target shapes under complex backgrounds, as it effectively reduces computational cost and memory usage while maintaining robust global context modeling capability.

**Figure 4:**
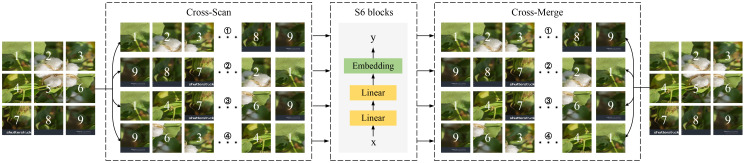
SS2D module.

As a core module of the model, SS2D consists of three key operations: Scan Expanding, the S6 module, which consists of S4 models with a selection mechanism and scan-based feature extraction, and Scan Merging. Unlike text data, which naturally possesses sequential characteristics, images lack inherent order. To address this, SS2D employs a scan expanding operation that unfolds the input image into a series of sub-images, as illustrated in **Figure 4**. Each sub-image represents a specific direction, and scanning is performed along four symmetrical orientations: top-to-bottom, bottom-to-top, left-to-right, and right-to-left. This approach not only ensures comprehensive coverage of all regions within the input image but also introduces directional variations, thereby enhancing the efficiency and completeness of multi-dimensional feature capture.

(1) $$Y_{d}=S6(X_{d}),d\in \{horizontal,vertical\}$$

Subsequently, the four generated one-dimensional vectors are fed into the S6 module for feature extraction, as shown in Equation 1. The S6 module incorporates a selective mechanism that ensures the dynamic adaptability of the weighting process, enabling the model to distinguish and retain relevant information while filtering out irrelevant features. By allowing each element of the one-dimensional vectors to interact with previously scanned information, the S6 module effectively captures diverse features and reduces the quadratic computational complexity to a linear level. Finally, the subgraphs are integrated through a scan merging operation to produce an output image consistent in size with the input.

The SS2D module not only possesses superior global semantic modeling capability but also better preserves the fine-grained structure of the target, while achieving a higher cost-effectiveness in terms of computational efficiency. Therefore, it is well-suited for cotton segmentation tasks under complex background conditions, demonstrating enhanced adaptability and stability.

### 3.3 ASPP module

Cotton exhibits a wide range of sizes and possesses irregular, complex, and fine-grained edge structures, posing significant challenges for precise segmentation. Traditional CNNs are limited by fixed receptive fields, making it difficult to effectively capture multi-scale contextual information; consequently, critical details may be lost when dealing with complex boundaries and fine textures. The ASPP module (Chen et al., 2017) employs multiple parallel convolutional branches with different dilation rates to perform multi-scale processing on the feature maps. In addition, the ASPP module includes an image-level global average pooling branch to provide richer global semantic context. Finally, by concatenating and fusing the outputs of all branches, ASPP achieves multi-scale feature enhancement while maintaining spatial resolution, thereby significantly improving the model’s segmentation capability for complex targets.

**Figure 5:**
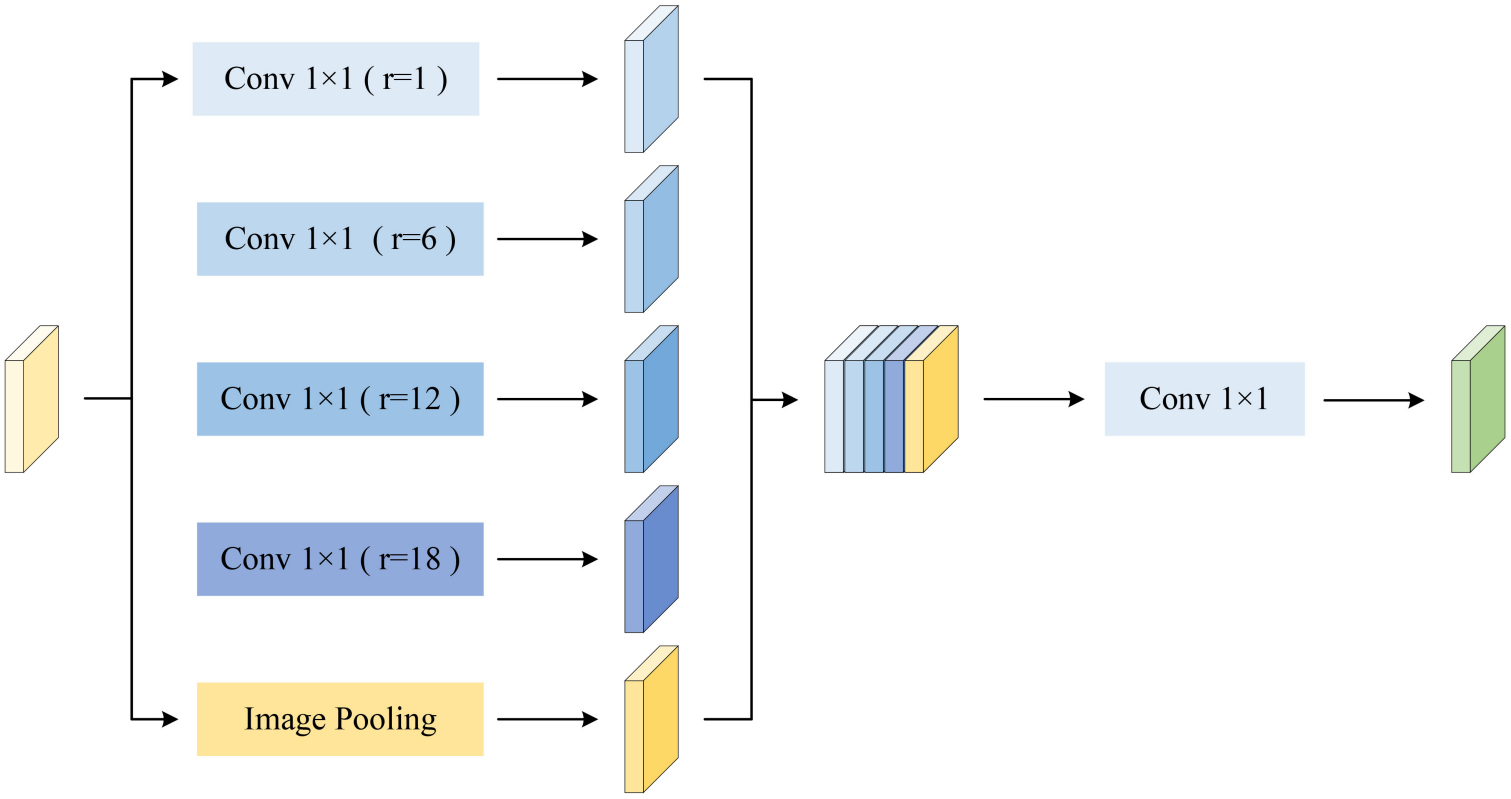
ASPP module.

Although the feature maps obtained from the CNN-branch encoder through multiple convolutional layers and downsampling contain rich semantic information, their receptive fields are relatively limited, making it difficult to fully capture the contextual information of targets at different scales. Therefore, an ASPP module is introduced at the end of the CNN branch, employing convolutional kernels with different dilation rates for feature extraction, as shown in Equation 2. Simultaneously, a global average pooling branch is employed to capture global contextual information. After being mapped back to the original dimension through a 1×1 convolution, the output of this branch is fused with the outputs of the other branches to form the final feature representation. As illustrated in **Figure 5**, by extracting and fusing multi-scale features in parallel, the model significantly enhances its sensitivity to fine cotton edges and complex characteristics, thereby more accurately distinguishing cotton from non-target regions. This effectively improves the network’s robustness under complex conditions and ensures high segmentation performance.

(2) $$y(\text{i})=\underset{k}{\sum }x\;(i+r\cdot k)\cdot w\;(k)$$

In Equation 2, y(i) represents the value at the i−th position in the output feature map; x(·) denotes the input feature map; w(k) refers to the convolution kernel weight at the k−th position; r is the dilation rate used to control the sampling interval of the convolution kernel; k is the index of the convolution kernel, typically ranging from -1 to 1; and x(i+r·k) indicates the selection of points from the input feature map in a skip manner.

### 3.4 DCNv1 module

The sampling positions of each convolution kernel in traditional convolution operations are fixed. However, the shapes of cotton are often irregular, and the cotton in images may exhibit deformation, distortion, and variations in scale. Traditional convolution is limited in capturing these variations and is less effective in learning critical shape features when processing edge details, thereby reducing segmentation accuracy.

**Figure 6:**
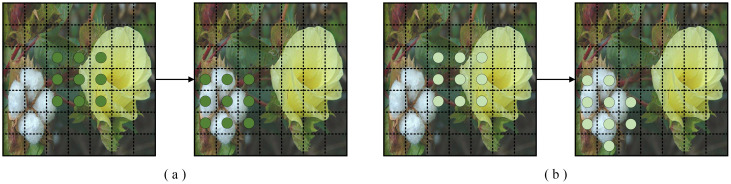
An example of convolution, where **(a)** is standard convolution and **(b)** is DCNv1 (Dai et al., 2017).

DCNv1 (Dai et al., 2017) is an improvement over traditional convolution, designed to enhance the handling of complex geometric shapes and highly deformable images. The core idea is to incorporate learnable offsets into the traditional convolution operation, allowing the convolution kernels to freely adjust their sampling locations within a local region rather than being fixed to regular grid positions, as illustrated in **Figure 6**. This enables the network to adaptively select more representative feature points for extraction, thereby improving the model’s robustness to variations in target shapes.

For each position in the input image, the convolution operation is defined as shown in Equation 3:

(3) $$y(p_{0})=\underset{p_{n}\in R}{\sum }w(p_{n})\cdot x(p_{0}+p_{n}+\text{Δ}p_{n})$$

where y $\;y(p_{0})$ represents the value of the output feature map at position $p_{0}$; x(·) refers to the input feature map; $w(p_{n})$ is the weight of the convolution kernel at position $p_{n}$; *R* denotes the sampling region of the standard convolution kernel; $p_{n}$ is a standard convolution offset at central position $p_{0}$; and $\text{Δ}p_{n}$ is the learned offset. In this way, the convolution operation can be conducted within a more flexible receptive field, adapting to the cotton present in the image.

### 3.5 scSE module

**Figure 7:**
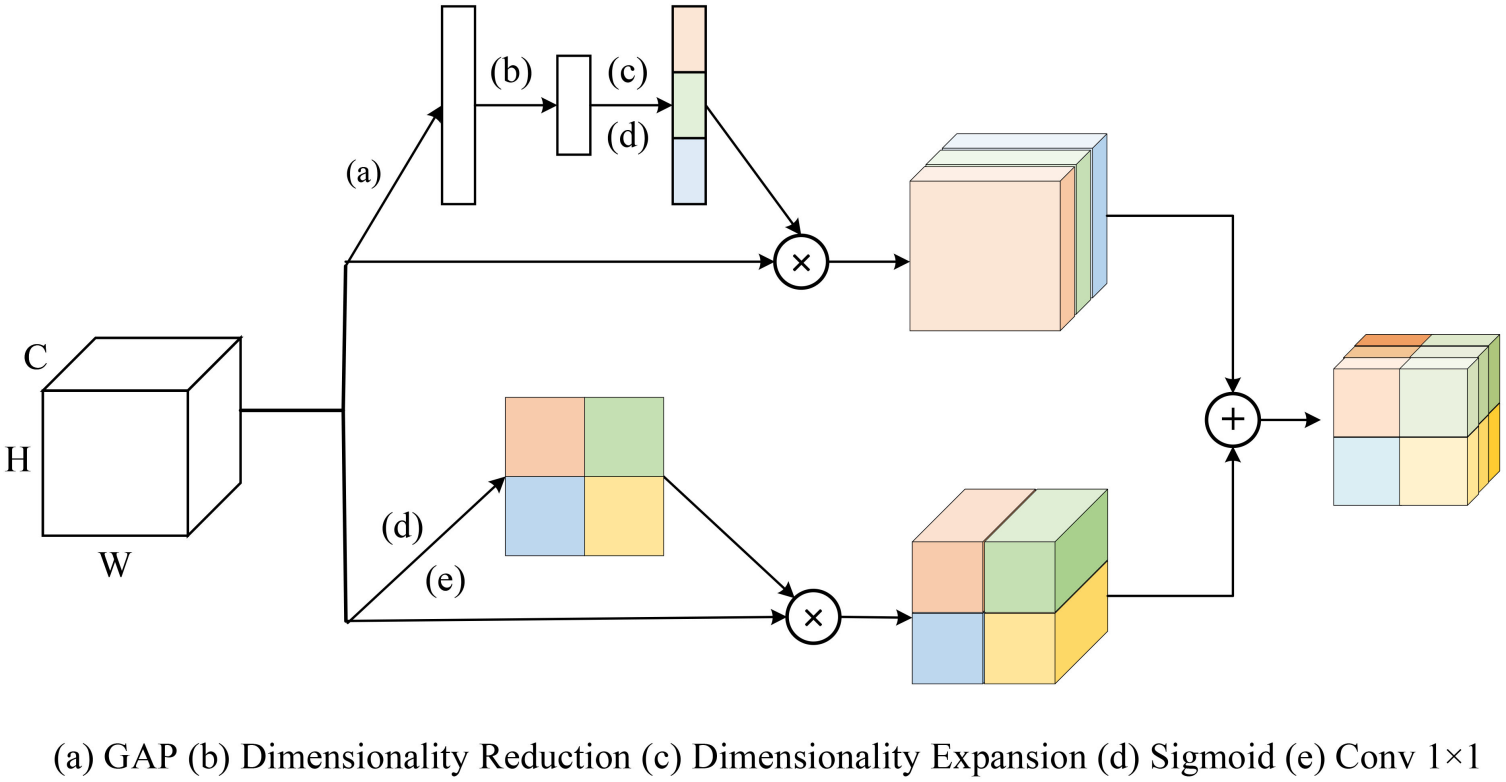
scSE module (Roy et al., 2018).

Due to the irregular morphology of cotton, this study employs a dual-branch architecture to simultaneously extract global information and local details. During the feature fusion stage, the outputs of both branches are combined to fully leverage the multi-scale features extracted by each branch, highlighting critical information in the target regions while suppressing background interference. However, directly fusing features from different branches through concatenation, addition, or averaging has several limitations. First, direct fusion lacks a selective mechanism to evaluate the importance of different features for the current task, which can easily introduce redundancy or even noise. In addition, feature maps from different branches may differ in semantic representation, spatial dimensions, or channel distribution, and direct fusion can result in information loss. The ParaTransCNN model employs the SE module (Hu et al., 2018) as a feature fusion component, which explicitly models inter-channel dependencies through a squeeze-and-excitation mechanism to enhance the network’s attention to key features. Nevertheless, the SE module does not sufficiently account for spatial dimension information, potentially leading to imprecise spatial localization of target regions. Therefore, in this study, the SE module is replaced with the scSE module (Roy et al., 2018). As illustrated in **Figure 7**, the scSE module introduces two parallel submodules, cSE and sSE, to achieve simultaneous feature recalibration across both channel and spatial dimensions.

Specifically, the cSE module primarily operates along the channel dimension. It compresses the input feature map $X\in R^{C\times H\times W}$ across the spatial dimensions through Global Average Pooling (GAP), thereby obtaining a global feature vector along the channel dimension. The specific computational process is shown in Equations 4–9.

(4) $$F_{GAP}\;(X)=\frac{1}{H\times W}\sum_{i=1}^{H}\sum_{j=1}^{W}X(i,j)$$

Subsequently, the vector is processed through two fully connected layers, initially reducing the dimensionality to decrease the computational load, followed by an increase in dimensionality to restore the number of channels. Ultimately, channel weights are obtained through normalization using the Sigmoid function:

(5) $$S_{\text{c}}(X)=\sigma \;(W_{2}\delta \;(W_{1}F_{GAP}\;(X)))$$

where $W_{1}$ and $W_{2}$ are two fully connected layers, r is the channel compression ratio, δ(·) denotes the ReLU activation function, and σ(·) signifies the Sigmoid normalization. Ultimately, channel attention is applied to the original feature map:

(6) $$X_{cSE}=S_{c}(X)\cdot X$$

The sSE submodule enhances salient regions by extracting attention weights along the spatial dimension through convolution.

(7) $$S_{s}(X)=\sigma \;(W_{s}\;*\;X)$$

where $W_{s}$ is a 1×1 convolution kernel, while ∗ represents the convolution operation. This weight is then multiplied by each element of the original feature map, which enhances the salient regions while suppressing the less important spatial regions.

(8) $$X_{sSE}=S_{s}(X)\cdot X$$

Finally, the scSE module integrates the outputs of both submodules, achieving more refined and comprehensive feature recalibration without altering the input dimensions.

(9) $$X_{scSE}=X_{cSE}+X_{sSE}$$

The cSE module focuses on enhancing global features, while the sSE module emphasizes local spatial information. Introducing the scSE module during the feature fusion stage not only strengthens the model’s ability to recognize target regions but also reduces background interference, thereby achieving more accurate segmentation results.

## 4 Dataset and experimental environment

### 4.1 Dataset

A portion of the image data in this study was obtained from the Roboflow platform (Luffy24312, 2023), which includes four major cotton varieties: Gossypium arboreum, Gossypium barbadense, Gossypium herbaceum, and Gossypium hirsutum, totaling 404 images. To enable the precise identification of high-quality cotton, the dataset was specifically optimized. First, low-quality cotton samples such as withered or decayed bolls were manually removed from the dataset to eliminate interference from non-target features, allowing the model to focus on learning the key morphological characteristics of high-quality cotton, such as boll fullness and fiber fluffiness. Second, additional cotton images captured under diverse conditions were incorporated, including samples taken at multiple scales, from different viewing angles, and under varying lighting conditions, as well as images featuring complex backgrounds—such as scenes containing branches, leaves, and weeds. This enhancement further improves the model’s robustness and discriminative capability in real-world environments, effectively increasing its accuracy in identifying mature cotton.

**Figure 8:**
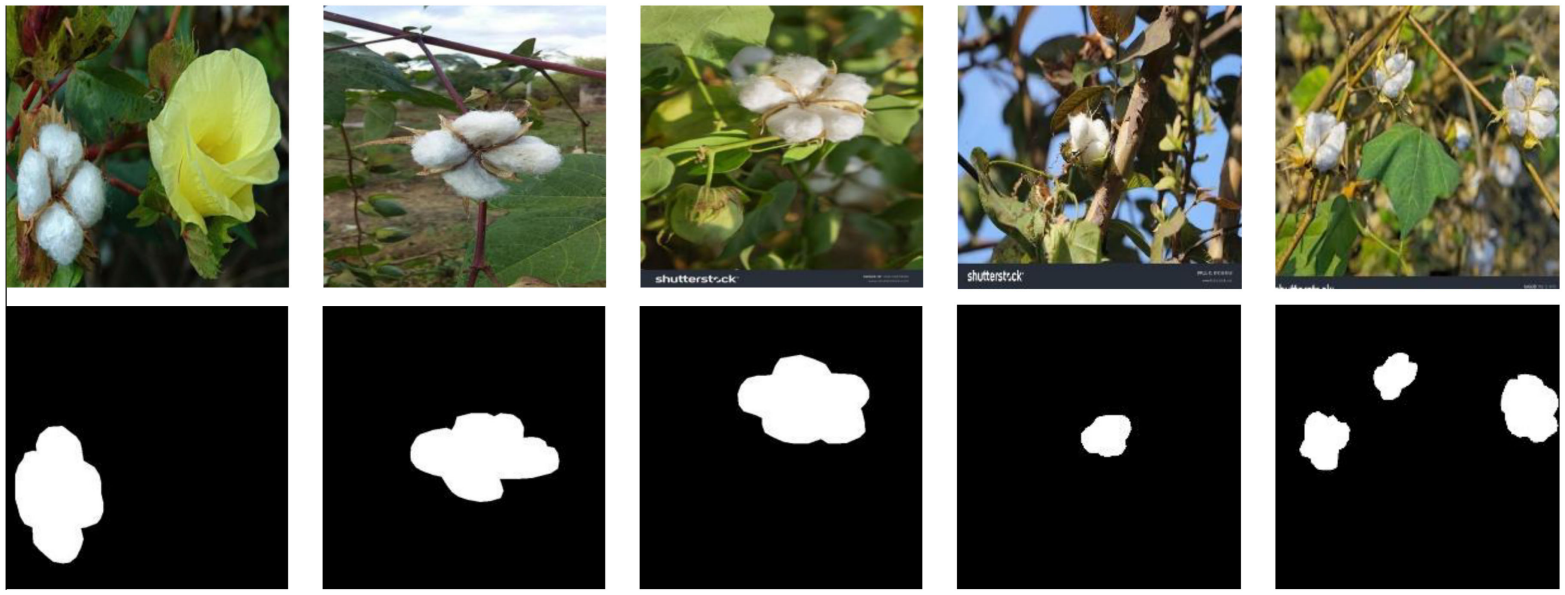
Data transformation process.

As shown in **Figure 8**, to ensure the consistency and scientific validity of the annotations, a unified labeling standard was established based on cotton morphological knowledge and existing annotated samples as references. All images were mask-labeled through the Roboflow platform and uniformly resized to 224×224 pixels. The dataset was divided into a training set and a test set at a ratio of 9:1.

Given that the processed cotton dataset in this study contains only 214 samples, the limited data size may constrain the model’s ability to capture the diversity of real-world scenarios and reduce its generalization performance during training. To address this issue, multiple data augmentation techniques were applied to the original images, including rotation, flipping, brightness and saturation adjustment, and noise addition. In addition, an improved Mosaic data augmentation method (Li et al., 2025) was introduced. Specifically, four images were randomly selected, and each was adaptively enhanced and cropped to half of its original height and width. Then, a 2×2 grid was used for random offsetting and concatenation to generate a Mosaic image. This method further improves the model’s adaptability to multi-scale dense objects and complex backgrounds. Ultimately, the dataset was expanded to 1,558 images, effectively increasing the diversity of training samples and simulating real-world conditions such as lighting variations, perspective deviations, and environmental noise.

### 4.2 Experimental setup

In order to ensure that the experimental results are as fair and effective as possible, all models were compared on the same standard experimental platform. With PyTorch as the deep learning framework and ParaTransCNN as the benchmark model, an experimental environment was created on a computing cloud platform. Its specific configuration is presented in **Table 1**. Meanwhile, the same hyperparameter settings were used in all the comparative models, as detailed in **Table 2**.

**Table 1:** Experimental environment configuration.

Environmental configuration	Value
Operating system	Ubuntu 22.04.4 LTS
GPU	NVIDIA GeForce RTX 4090 D
CPU	AMD EPYC 9754 CPU @ 3.10GHz
Programming language	Python 3.12.3
Deeplearning framework	PyTorch2.5.1+cu124
CUDA	CUDA12.4

**Table 2:** Hyperparameter configuration.

Hyperparameter	Value
LearningRate	0.01
ImageSize	224×224
Optimizer	SGD
Momentum	0.9
BatchSize	4
Epoch	50

### 4.3 Evaluation metrics

In order to comprehensively evaluate the performance of the models in the cotton segmentation task, six evaluation metrics were used to reflect the model performance in terms of precision and complexity from multiple perspectives. The specific computational process is shown in Equations 10–13.

Dice coefficient (Dice) measures the overlap between the predicted segmentation region and the ground truth annotation.

(10) $$D\text{ice}=\frac{2\times |A\cap B|}{\left|A|+|B\right|}=\frac{2\times TP}{2\times TP+FP+FN}$$

where *A* represents the predicted segmentation region, *B* represents the ground truth annotation, *TP* denotes the number of true positives, *FP* denotes the number of false positives, and *FN* denotes the number of false negatives.

95% Hausdorff Distance (HD95) measures the maximum geometric error between the predicted boundary and the ground truth boundary. By excluding 5% of outliers, this metric effectively reduces sensitivity to noise.

(11) HD95(A,B)=max{95thpercentile (d (a,B)),95thpercentile (d (b,A))}

where *d*(*a,B*) represents the shortest Euclidean distance from point *a* to set *B*, and vice versa.

Mean Intersection over Union (mIoU) comprehensively reflects the overall segmentation accuracy and is calculated as the average of the IoU values across all classes.

(12) $$mIoU=\frac{1}{C}\sum_{i=1}^{C}\frac{TP_{i}}{TP_{i}+FP_{i}+FN_{i}}$$

where *C* denotes the total number of classes, with two classes in this study, and *i* denotes the class index. Pixel Accuracy (Accuracy) measures the ratio of correctly classified pixels to the total number of pixels.

(13) $$A\text{ccuracy}=\frac{TP+TN}{TP+TN+FP+FN}$$

Where *TN* represents the number of true negatives.

In addition, this study measures the model’s complexity and computational cost using Params and Giga Floating Point Operations Per Second (GFLOPS).

## 5 Results and analyses

### 5.1 Comparative experiment with mainstream models

To comprehensively evaluate the segmentation performance of the proposed model, a comparative study was conducted against representative mainstream methods, including ParaTransCNN (Sun et al., 2024), SwinUNet (Cao et al., 2022), TransUNet (Chen et al., 2021), TransCeption (Azad et al., 2023b), TransDeepLab (Azad et al., 2022), MissFormer (Huang et al., 2022), HiFormer (Heidari et al., 2023), and DAEFormer (Azad et al., 2023a). All models were trained and tested under identical experimental conditions.

**Figure 9:**
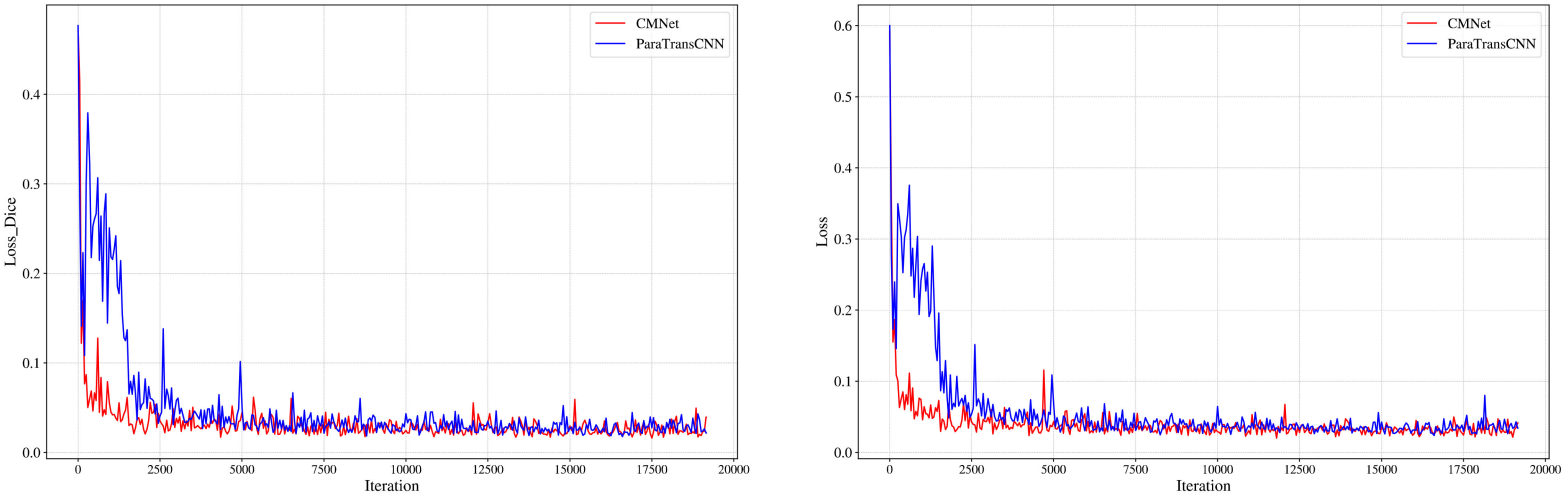
Comparison in model loss function.

As shown in **Table 3**, the proposed model outperforms ParaTransCNN across all evaluation metrics. The Dice coefficient reaches 91.06%, higher than ParaTransCNN’s 88.58%, indicating a greater overlap between the predicted segmentation regions and the ground truth annotations. In terms of boundary localization, the HD95 of our model is 2.47, significantly lower than that of ParaTransCNN, demonstrating that the improvements enable more precise capture of fine cotton structures and effectively reduce boundary errors. In addition, the mIoU and Accuracy of the proposed model are improved by 3.05% and 0.29%, respectively, compared with the baseline model. These results indicate that, based on ParaTransCNN, the proposed model enhances the ability to model edge variations and achieves more effective integration of global and local features, thereby improving performance in capturing irregular shapes and complex edges. This fully validates the superiority of the improved model in precise cotton segmentation tasks. As shown in **Figure 9**, the proposed model exhibits a faster convergence rate and maintains a lower loss value during training, further demonstrating its efficiency and stability in the optimization process, which enables it to achieve satisfactory segmentation performance within a shorter training time.

**Table 3:** Results of comparative experiment with other models.

Model	Dice (%)	HD95	mIoU (%)	Accuracy(%)
ParaTransCNN (Sun et al., 2024)	88.58	7.56	81.13	97.81
SwinUnet (Cao et al., 2022)	85.84	12.84	**90.49**	97.50
TransUnet (Chen et al., 2021)	89.19	11.74	81.54	97.74
TransCeption (Azad et al., 2023b)	85.21	13.28	77.45	97.33
TransDeepLab (Azad et al., 2022)	83.87	14.70	75.41	97.00
MissFormer (Huang et al., 2022)	83.35	16.21	75.17	96.74
HiFormer (Heidari et al., 2023)	89.18	7.89	81.54	97.67
DAEFormer (Azad et al., 2023a)	82.89	9.47	74.54	96.76
YOLOv8seg	80.27	25.51	69.93	93.08
Ours	**91.06**	**2.47**	84.18	**98.10**

Bold values indicate the best performance.

**Figure 10:**
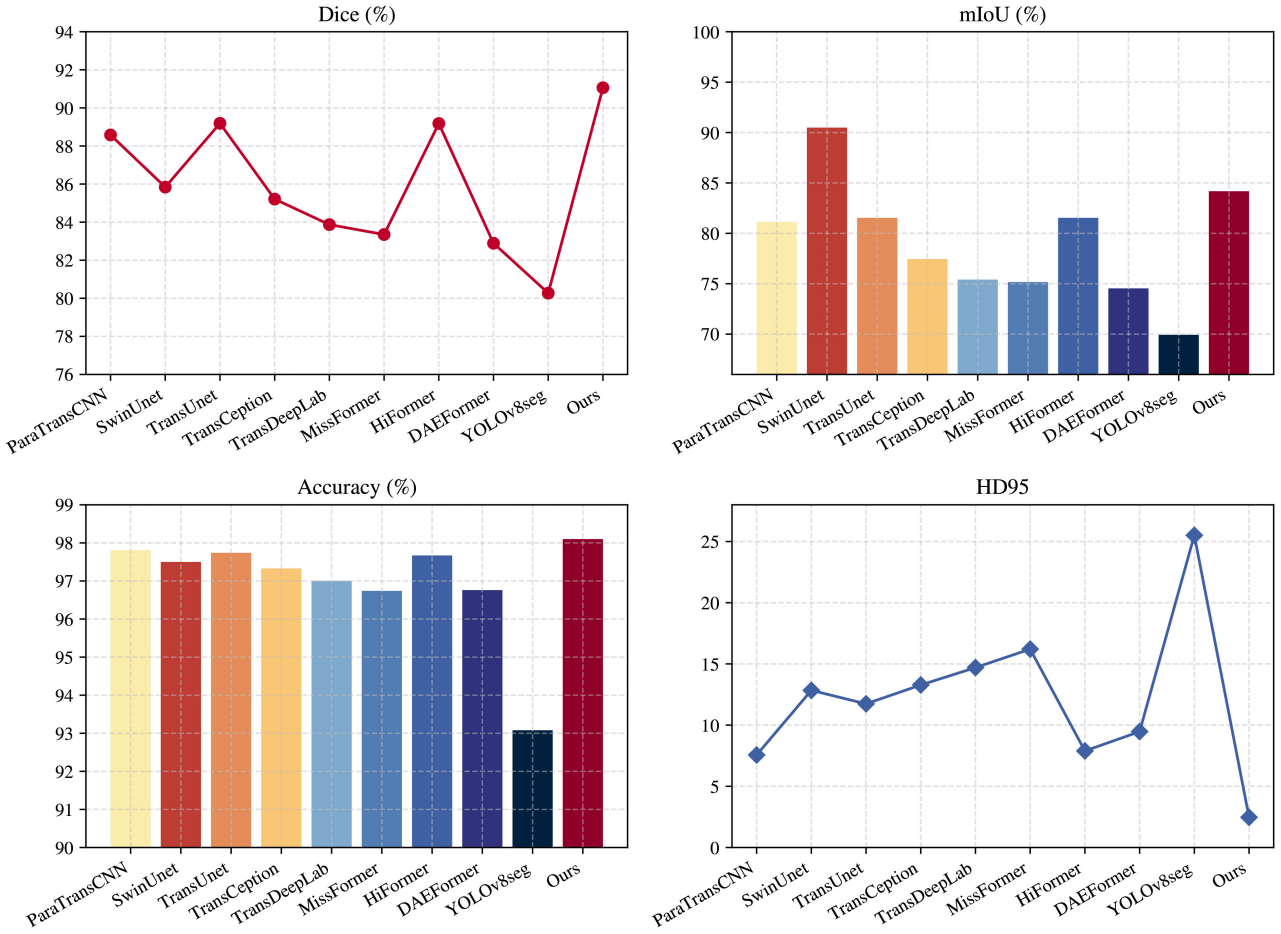
Comparison of performance among models.

As shown in **Figure 10**, compared with other mainstream segmentation models, the proposed model achieves a significantly higher Dice coefficient than Transformer-based or hybrid models such as HiFormer and TransCeption, indicating a higher degree of consistency and overlap between the predicted segmentation regions and the ground truth annotations. This improvement can be largely attributed to the SS2D module, which effectively enhances the model’s global modeling capability. Although models such as MissFormer and TransDeepLab demonstrate good overall accuracy, their HD95 values are noticeably higher, indicating the presence of certain boundary localization errors. In contrast, the introduction of the deformable convolution module DCNv1 in our model effectively enhances its ability to model irregular boundary variations in cotton, allowing adaptive adjustment of convolution sampling positions to more accurately capture complex edges and fine-grained details. As a result, the HD95 value is significantly lower than that of other models. The SwinUNet model exhibits relatively high performance in mIoU and Accuracy; however, its Dice coefficient and HD95 are significantly inferior to those of the proposed model, indicating that its ability to segment fine-grained targets remains insufficient.

Except for the proposed model, TransUnet performs relatively well, achieving a Dice coefficient of 89.19%. However, due to its lack of adaptive modeling capability for local details, this model still exhibits noticeable deficiencies in boundary delineation. Among all compared models, YOLOv8-Seg demonstrates the weakest performance, showing clear limitations in handling cotton segmentation. This is primarily because YOLOv8-Seg prioritizes detection speed and end-to-end inference efficiency, resulting in relatively shallow feature extraction depth, which leads to blurred boundaries and frequent missed detections in complex cotton scenes. Overall, the proposed model not only maintains high accuracy but also significantly enhances boundary localization and global consistency, fully demonstrating its advantages in complex cotton segmentation tasks.

### 5.2 Ablation experiments

To evaluate the impact of the proposed improvements on cotton segmentation modeling, a series of ablation experiments were designed based on the ParaTransCNN baseline model. Different modules were progressively replaced or removed to assess their influence on model performance, thereby clarifying the specific contribution of each improvement to the overall performance of the model.

As shown in **Table 4**, incorporating the SS2D module into the baseline model significantly improved its global modeling capability, resulting in notable increases in Dice and mIoU, reaching 89.82% and 82.30%, respectively. In addition, both the computational cost and the number of parameters slightly decreased, indicating that SS2D effectively reduces computational burden while maintaining high accuracy. The ASPP module, with its parallel atrous convolutions, effectively expands the receptive field, enabling the model to capture stronger multi-scale feature representations while preserving high-resolution details. Experimental results show that although the Dice coefficient slightly decreased after introducing ASPP, other metrics improved, with HD95 reduced to 2.86, indicating a significant enhancement in the model’s ability to recover fine-grained details. When the scSE module was applied independently, all performance metrics slightly declined, suggesting that it cannot fully realize its potential without interaction with other modules. Moreover, incorporating DCNv1 alone led to minor improvements in Dice and mIoU, but HD95 increased from 7.56 to 8.83, indicating instability in boundary prediction. This suggests that while DCNv1 effectively models local geometric deformations, it lacks guidance from global semantic structures and is therefore prone to local optima.

**Table 4:** Results of ablation experiments.

SS2D	ASPP	scSE	DCNv1	Dice(%)	HD95	mIoU(%)	Accuracy(%)	Params(M)	GFLOPs
				88.58	7.56	81.13	97.81	245.5	1136
✓				89.82	6.63	82.30	97.86	206.8	1101
	✓			87.99	2.86	81.55	98.04	233.2	1130
		✓		88.28	10.18	80.14	97.41	249.2	1136
			✓	88.98	8.83	81.34	97.73	245.8	1137
✓	✓			90.45	5.65	83.31	97.99	**194.5**	**1095**
✓	✓	✓		90.79	**2.45**	84.04	**98.14**	197.5	1096
✓	✓	✓	✓	**91.06**	2.47	**84.18**	98.10	197.8	1096

Bold values indicate the best performance.

**Figure 11:**
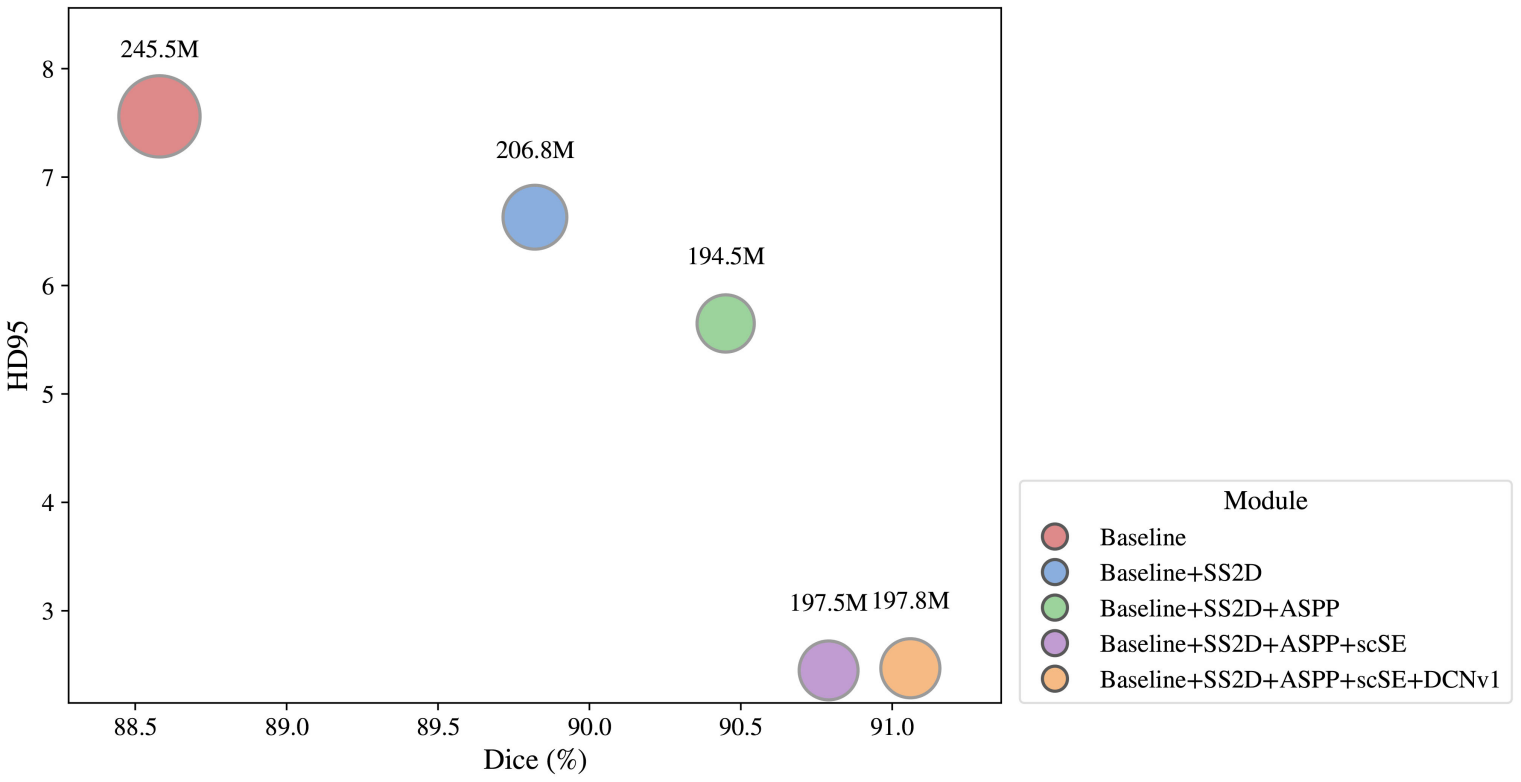
Performance of the model after superposition of modules.

Therefore, this study integrates the SS2D, DCNv1, ASPP, and scSE modules to construct a comprehensive model. As illustrated in **Figure 11**, the combination of these modules exhibits a significant synergistic effect.

With the inclusion of the ASPP module on top of the SS2D module, the model effectively aggregates multi-scale contextual information, substantially reducing boundary errors, with the HD95 decreasing to 2.86. This indicates stronger robustness under complex field conditions. The scSE module adaptively recalibrates features along both the spatial and channel dimensions, further strengthening the representation of key information and improving both Dice and mIoU metrics. Meanwhile, the DCNv1 module enhances the model’s ability to capture irregular cotton contours, contributing to more accurate boundary localization. When all four modules are combined, they demonstrate a remarkable synergistic effect: the complete model achieves the highest performance across all four metrics while maintaining relatively low parameter and computational complexity, with the Dice coefficient improving by 2.48 percentage points. These results indicate that the modules complement each other, mitigating issues of local optimization and error propagation, effectively enhancing the model’s representational capacity and generalization performance, and providing reliable technical support for intelligent cotton recognition and segmentation in complex natural environments.

### 5.3 Comparative experiments of the ASPP module

In image segmentation tasks, accurately perceiving the morphology and scale of targets is a crucial factor for enhancing model performance. Therefore, this study selected several representative multi-scale structural modules, including Spatial Pyramid Pooling (SPP) (He et al., 2015), SPPF, SimSPPF, Receptive Field Block (RFB) (Liu and Huang, 2018), SPPCSPC (Wang et al., 2023), SPPCSPCgroup, SPPFCSPC (Li et al., 2023), and ASPP (Chen et al., 2017), to conduct a comparative analysis of their performance in cotton segmentation tasks. The quantitative comparison results are summarized in **Table 5**. This comparison aims to evaluate the differences among various structures and provide both theoretical and practical guidance for model architecture design.

**Table 5:** Comparison of different spatial pyramid modules.

Model	Dice (%)	HD95	mIoU (%)	Accuracy(%)
SPP (He et al., 2015)	90.66	2.60	83.62	98.06
SPPF	90.37	2.87	83.22	98.06
SimSPPF	89.68	2.79	82.85	98.08
RFB (Liu and Huang, 2018)	87.16	5.24	79.91	97.84
SPPCSPC (Wang et al., 2023)	89.74	4.62	82.46	97.97
SPPCSPCgroup	90.23	2.55	83.16	98.04
SPPFCSPC (Li et al., 2023)	90.76	4.28	83.88	98.09
ASPP (Chen et al., 2017)	**91.06**	**2.47**	**84.18**	**98.10**

Bold values indicate the best performance.

**Figure 12:**
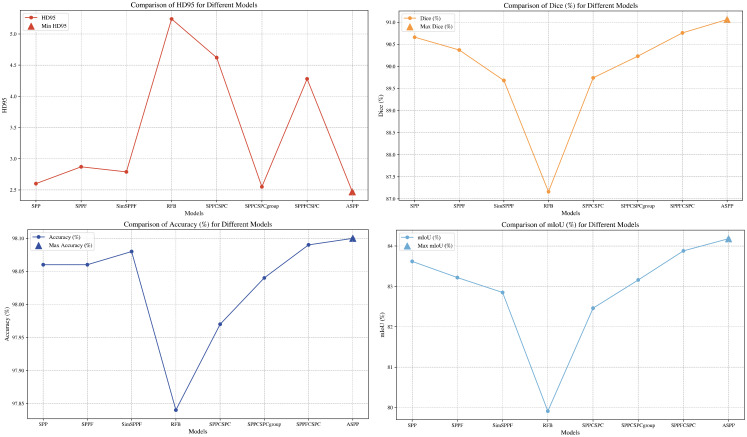
Comparison of metrics across modules.

As shown in **Figure 12**, the ASPP module demonstrated the best overall performance, highlighting its efficiency in capturing multi-scale features. The SPPFCSPC module also performed well, ranking just below ASPP in Dice and mIoU, with an accuracy of 98.09%; however, its relatively high HD95 indicates room for further improvement in boundary regions. The SPPF and SimSPPF modules showed slight declines in accuracy, particularly SimSPPF, suggesting that excessive simplification may negatively affect segmentation precision. In contrast, the RFB module performed the worst among all structures, achieving a low Dice score and a high HD95, possibly due to its spatial selection mechanism being unsuitable for the diverse cotton targets.

### 5.4 Comparative experiments of different convolutional modules

To further investigate the impact of different types of convolutions in the VMamba branch on model performance, a series of comparative experiments were conducted, including Depthwise Convolution (DWConv) (Chollet, 2017), Dilated Conv (Yu and Koltun, 2015), Dynamic Conv (Chen et al., 2020), GhostConv (Han et al., 2020), and various variants of deformable convolutions. The model was systematically evaluated across multiple metrics, and the results are presented in **Table 6**.

**Table 6:** Comparison of different convolutional modules in the VMamba branch.

Model	Dice (%)	HD95	mIoU (%)	Accuracy(%)
DWconv (Chollet, 2017)	90.82	3.66	83.76	97.97
Dilated Conv (Yu and Koltun, 2015)	89.41	4.45	82.13	97.73
Dynamic Conv (Chen et al., 2020)	90.07	5.56	82.93	98.04
Ghostconv (Han et al., 2020)	87.35	5.67	80.94	97.99
DCNv2 (Zhu et al., 2019)	90.17	3.00	82.94	98.03
DCNv3	64.44	28.87	52.84	92.61
DCNv4 (Xiong et al., 2024)	90.53	5.64	83.50	98.06
DCNv1	**91.06**	**2.47**	**84.18**	**98.10**

Bold values indicate the best performance.

**Figure 13:**
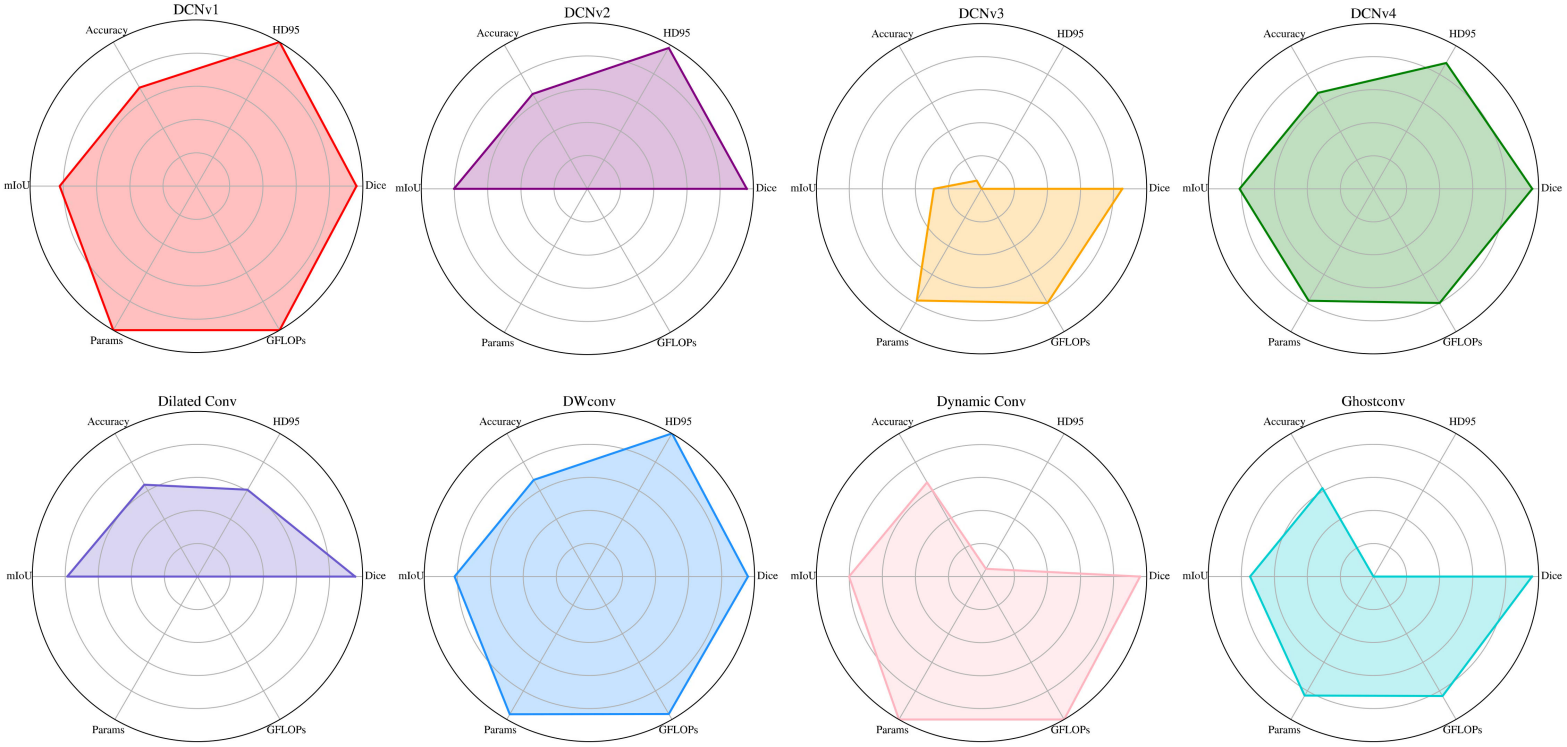
Radar charts of different convolutions.

As shown in **Figure 13**, the radar charts utilize six-dimensional axes to comprehensively display the differences of each module across multiple metrics, facilitating an overall assessment of their adaptability and practicality. Among all compared modules, DCNv1 demonstrated the best performance, exhibiting outstanding region overlap and boundary-capturing capability. DCNv2 and DCNv4 also showed strong modeling ability, maintaining high levels across multiple metrics, indicating good potential for adapting to complex target shapes. However, DCNv3 performed significantly worse than the other modules in this task, likely due to its complex parameter scheduling mechanism, which is not suitable for small targets, resulting in poor boundary handling and region overlap. Additionally, DWConv achieved a good balance, with Dice and mIoU values close to those of DCNv1. In contrast, GhostConv exhibited notably insufficient segmentation performance, particularly with substantial declines in Dice and HD95, making it unsuitable for modeling complex spatial relationships. Dilated Conv maintained a certain receptive field at the cost of boundary precision, while Dynamic Conv improved representational capacity to some extent but suffered from higher boundary errors, limiting its overall performance enhancement.

### 5.5 Comparative experiments of feature fusion modules

To evaluate the impact of different feature fusion modules on the performance of cotton segmentation models, this study conducted multiple experiments. These modules can be categorized into three types based on their attention dimensions. Channel attention modules, such as SE (Hu et al., 2018), Efficient Channel Attention (ECA) (Wang et al., 2020), and CA (Hou et al., 2021), primarily model the weight relationships between channels, emphasizing key feature channels to enhance global representation capacity. Spatial attention modules, such as Large Kernel Attention (LKA) (Guo et al., 2023), model response regions along the spatial dimension, guiding the network to focus on important locations within the image, thereby improving boundary localization accuracy. Hybrid attention modules, such as Convolutional Block Attention Module (CBAM) (Woo et al., 2018), Bottleneck Attention Module (BAM) (Park et al., 2018), scSE (Roy et al., 2018), and Global Attention Mechanism (GAM) (Hu et al., 2023), integrate both channel and spatial mechanisms, enabling a balance between local detail and global structural information, thereby collaboratively optimizing feature selection and information representation capabilities.

As shown in **Table 7**, the ECA module performed well, demonstrating its superiority in channel feature recalibration and information extraction, though it was still slightly inferior to the scSE module. The segmentation performance of the SE module is slightly inferior, with an mIoU of only 80.48%. The LKA module enhances attention to specific spatial regions through dynamic adjustment of local convolution kernels; however, its performance remains slightly insufficient, as evidenced by an HD95 as high as 9.62. These results indicate that channel attention modules primarily focus on recalibrating channel features, while spatial attention modules emphasize spatial responses in specific regions; when used individually, neither can effectively balance global structural information with local details, resulting in certain performance limitations.

**Table 7:** Comparison of different feature fusion modules.

Model	Dice (%)	HD95	mIoU (%)	Accuracy(%)
GAM (Hu et al., 2023)	88.32	10.96	80.53	97.75
CBAM (Woo et al., 2018)	89.85	6.34	82.41	97.85
CA (Hou et al., 2021)	89.51	4.78	82.13	97.59
ECA (Wang et al., 2020)	89.95	5.66	82.69	98.01
LKA (Guo et al., 2023)	89.71	9.62	82.07	97.71
BAM (Park et al., 2018)	89.85	4.18	82.51	97.72
SE (Hu et al., 2018)	87.50	7.20	80.48	97.83
scSE (Roy et al., 2018)	**91.06**	**2.47**	**84.18**	**98.10**

Bold values indicate the best performance.

Hybrid attention modules such as CBAM and BAM performed relatively well and comparably, achieving a Dice coefficient of 89.85%. However, since both modules primarily rely on a sequential attention computation mechanism, they may not fully capture the complex spatial dependencies present in cotton images, resulting in a slight performance gap compared to the scSE module. In contrast, the performance of the GAM module was inferior, likely due to its global attention mechanism being less precise in handling fine details, which can lead to blurred boundaries or inaccurate segmentation.

Overall, modules that employ a single attention mechanism exhibit limitations in capturing both feature information and spatial structures, whereas hybrid modules demonstrate a clear advantage in capturing fine-grained cotton features. Among them, the scSE module achieves the best performance; its parallel architecture adaptively recalibrates both spatial and channel dimensions, avoiding potential information bottlenecks associated with sequential attention and enabling complementary modeling of spatial saliency and channel importance. This design significantly enhances the model’s ability to represent fine-grained features and improves boundary segmentation accuracy.

**Figure 14:**
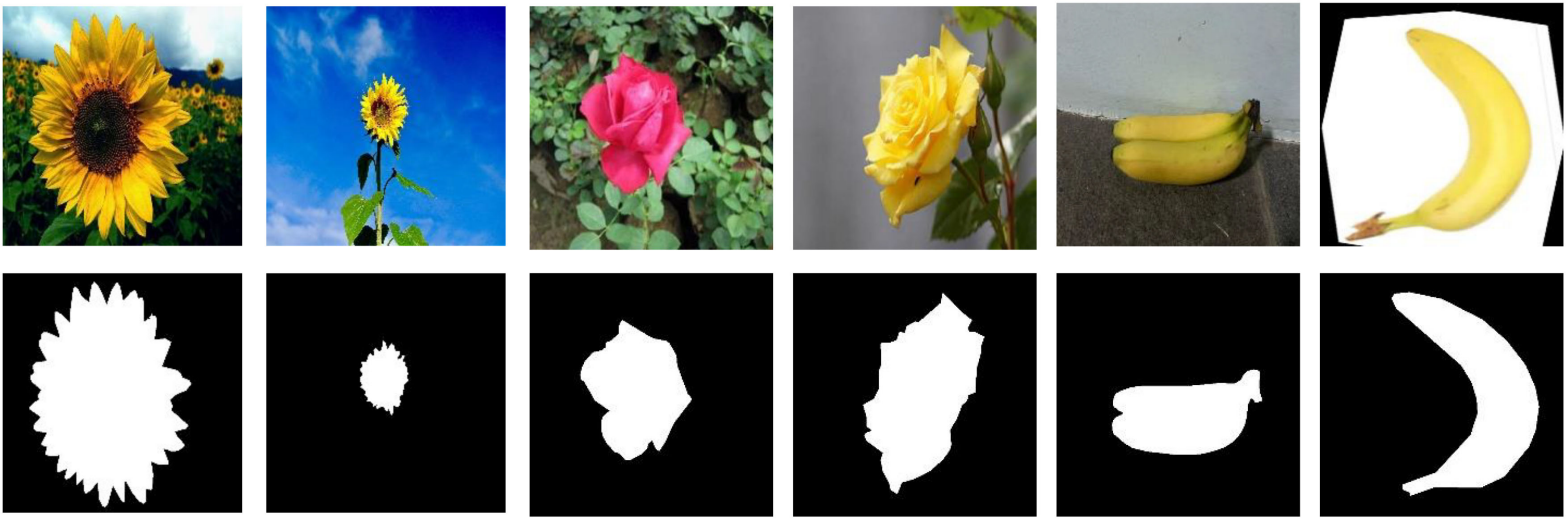
Sunflower data.

### 5.6 Generalization experiment

To evaluate the generalization capability of CMNet, this study utilized the Roboflow platform to select three agricultural datasets with distinct characteristics—sunflower (ELTE, 2023), rose (Vyuha, 2025), and banana (Laola, 2024). As shown in **Figure 4**, the sunflower dataset shares a high degree of similarity with cotton in terms of growth cycle and harvesting method, while also exhibiting complex texture structures. The rose dataset features pronounced morphological diversity and substantial background variations, making it suitable for assessing the model’s segmentation performance under complex environments. The banana dataset, characterized by slender shapes and regularly arranged fruits, was employed to examine the model’s adaptability to different crop structures and morphologies. By conducting generalization experiments on these three datasets, the adaptability of CMNet across various plant species can be systematically verified, thereby further demonstrating the model’s reliability in agricultural visual perception tasks.

As shown in **Table 8**, the CMNet model significantly outperforms ParaTransCNN across all three plant datasets, fully demonstrating its strong generalization capability. Specifically, on the sunflower dataset, CMNet achieves remarkable improvements in both Dice and mIoU metrics, indicating that the model can more effectively capture fine-grained textures and edge details of the complex floral structures. This further confirms that the introduction of DCNv1 enhances the model’s sensitivity to intricate structural features. On the rose dataset, CMNet maintains stable segmentation performance, with the Dice value improving by more than 20% compared to ParaTransCNN. This result suggests that the model effectively suppresses background interference and exhibits greater robustness when handling targets with irregular contours. For the banana dataset, CMNet achieves the lowest HD95 value, substantially lower than that of ParaTransCNN, indicating that the model maintains high segmentation accuracy across different geometric shapes.

**Table 8:** Segmentation performance of different datasets on ParaTransCNN and the proposed model.

Dataset	Model	Dice (%)	HD95	mIoU (%)	Accuracy (%)
Sunflower	ParaTransCNN Ours	81.17 **91.03**	3.60 **1.24**	71.88 **83.63**	95.68 **96.98**
Rose	ParaTransCNN Ours	67.36 **87.54**	27.13 **9.58**	54.83 **79.70**	82.21 **93.94**
Banana	ParaTransCNN Ours	78.95 **93.54**	10.31 **1.61**	69.71 **88.14**	89.15 **96.19**

Bold values indicate the best performance.

The three selected datasets exhibit significant differences in morphological characteristics and target structures, posing challenges for cross-crop segmentation and detection. However, CMNet consistently demonstrates superior performance across all datasets, which fully indicates that the integration of multiple advanced modules enables the model to effectively adapt to variations in plant features. This highlights CMNet’s strong cross-domain generalization capability and adaptability, providing robust technical support for intelligent and precision agriculture management.

### 5.7 Result visualization

**Figure 15:**
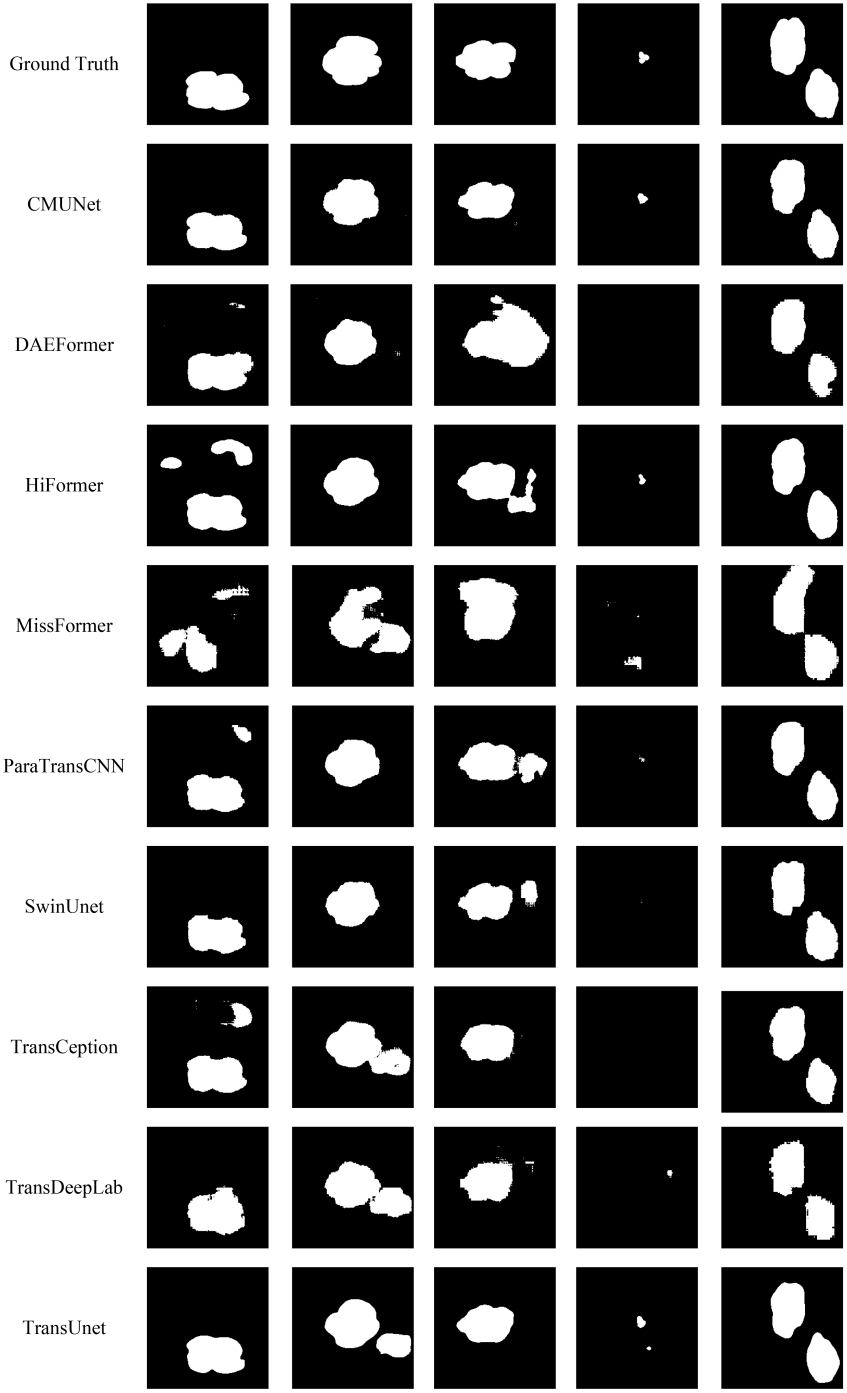
Comparison of visualized segmentation results of various models.

To more intuitively demonstrate the performance of the proposed CMNet model in cotton image segmentation, a comparative visualization of the prediction results from the segmentation models was conducted. The selected comparative models encompass all the models involved in the previous performance evaluations. **Figure 15** presents the segmentation results of the test images, with each column showing the ground truth and the predicted masks generated by each model, respectively.

As shown in **Figure 15**, CMNet performed the best among all comparative models. For all samples, the predicted masks generated by CMNet exhibited clear boundaries and complete structures, highly consistent with the ground truth labels. This model is capable of accurately extracting the contours of cotton regions while effectively suppressing background interference, demonstrating good segmentation robustness and boundary awareness. CMNet successfully avoided false detections while maintaining good recognition accuracy for small target regions, where other models often make errors. In contrast, MissFormer performed the worst, as the masks it generated not only suffered from significant over-segmentation issue but also frequently misidentified background regions as cotton, resulting in predictions that deviate far from the ground truth values, making the model less usable and reliable. With better performance, TransUnet exhibited certain advantages in recognizing medium-sized cotton targets. However, its segmentation boundaries remained blurred, resulting in some precision loss.

In addition, it can be observed that in the images in the second column, multiple models mistakenly identified background regions as cotton, resulting in significant false positive regions, which could lead to serious recognition errors in practical applications. Meanwhile, CMNet exhibited stable performance in this challenging scenario, accurately distinguishing between cotton and background regions, demonstrating superior feature differentiation capability and strong background suppression ability. The images in the fourth column further highlighted the differences in model performance concerning the segmentation of small targets, as several models failed to recognize the small-area cotton targets in the image, showing insensitivity to sparse targets. In contrast, CMNet effectively identified these small targets through the introduction of a multi-scale feature fusion mechanism, ensuring the integrity and accuracy of the segmentation results. The fifth column also illustrates that CMNet consistently achieves the best overall performance when segmenting multiple cotton bolls.

### 5.8 Application of the CLIP model in cotton-picking robots

Compared with traditional manual and single-mechanism harvesting methods, cotton-picking robots offer significant advantages in efficiency and accuracy. To this end, the CLIP model was introduced to construct a semantically enhanced robotic perception framework. CLIP is a multimodal pre-training model that maps images and text into a unified embedding space via contrastive learning, enabling cross-modal semantic alignment. Leveraging the powerful generalization capability of CLIP, segmented cotton region images along with their corresponding basic visual descriptions were jointly encoded through the CLIP encoder to generate semantic representation vectors aligned with the specific states of the cotton. These high-level semantic embeddings were subsequently decoded by a dedicated text decoder into structured instructions, facilitating accurate and context-aware decision-making by the robot.

**Figure 16:**
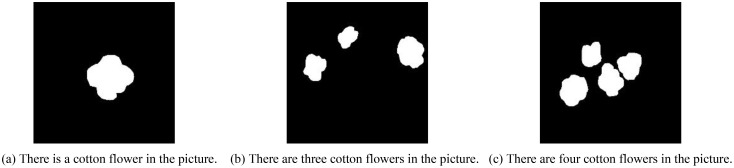
Utilizing the CLIP model to read image content.

As shown in **Figure 16**, the CLIP model can effectively interpret the content of images and accurately count the number of intact cotton bolls. This provides robots with more precise picking information, enabling them to make informed harvesting decisions, avoid mispicking or missing cotton bolls due to misjudgment, improve picking efficiency, and further advance the development of smart agriculture.

## 6 Discussion and limitations

### 6.1 Discussion

Previous studies on cotton segmentation have achieved significant progress. For instance, Tan et al. (2025) combined an improved DM-Count with the SAM segmentation model to predict cotton boll number, size, and seed cotton yield from aerial images. Although this approach demonstrated excellent segmentation performance, it still suffered from inaccuracies under complex conditions, small cotton bolls, or occlusions. Bolouri et al. (2024) developed the CottonSense system, which enabled 3D cotton segmentation and high-throughput phenotyping, providing breeders with valuable insights into crop growth patterns and developmental stages. However, the accuracy of this method was constrained by the Mask R-CNN model, limiting its segmentation effectiveness. Overall, existing approaches struggle to achieve irregular morphology perception in complex field environments.

In this study, we propose CMNet, an improved dual-branch network based on the ParaTransCNN architecture. The original Transformer branch is replaced with an SS2D module, which balances local detail extraction and global semantic representation while reducing computational overhead. This branch also integrates a DCNv1 module to enhance the perception of irregular cotton structures, while the CNN branch incorporates an ASPP module to strengthen multi-scale feature representation. Furthermore, the traditional SE module is replaced with the scSE attention mechanism, optimizing the fusion of channel and spatial features and improving overall feature modeling capability. Compared with previous methods, CMNet effectively overcomes their limitations, enabling accurate segmentation of irregularly shaped cotton under complex background conditions. These improvements not only enhance segmentation accuracy and robustness but also provide support for cotton growers in developing optimized management strategies.

### 6.2 Limitations

Despite the significant progress achieved by CMNet in cotton image segmentation, several limitations remain. First, the model’s performance may decline under extreme lighting conditions or severe occlusions.

Second, although CMNet demonstrates robust performance on cotton and a limited set of other crop datasets, its generalization capability across diverse crop structures and morphologies still requires further validation. Moreover, despite the satisfactory results obtained in the current generalization experiments, the limited dataset size may constrain the model’s ability to capture the full variability of cotton appearance and complex field conditions, potentially affecting its performance in highly heterogeneous agricultural scenarios. Future work will focus on expanding the dataset to include a wider range of crop species, growth stages, and environmental conditions, while also optimizing the network architecture to further enhance the model’s adaptability and robustness across multiple crop scenarios. Finally, although the current study has achieved a degree of model lightweighting based on the original architecture, the computational cost remains relatively high for practical deployment; therefore, future efforts will explore techniques such as knowledge distillation and pruning to reduce model complexity without compromising performance.

## 7 Conclusion

This study proposes an improved dual-branch cotton segmentation network, CMNet, designed to address challenges in cotton segmentation tasks such as background interference and irregular cotton morphology. Based on the ParaTransCNN architecture, CMNet replaces the Transformer module with the SS2D module, effectively reducing computational complexity. In addition, the model incorporates DCNv1 to enhance adaptability to complex cotton shapes, employs the ASPP module to improve multi-scale feature extraction, and optimizes feature fusion through the scSE module, further boosting overall model performance. Experimental results demonstrate that CMNet outperforms existing methods in key metrics such as Dice and HD95. Moreover, while maintaining segmentation accuracy, the model significantly reduces parameter count and computational cost. Generalization experiments indicate that CMNet achieves excellent segmentation performance and strong generalization capability across multiple other plant datasets, providing a reliable technical foundation for automated management and precise segmentation tasks in smart agriculture.

Nevertheless, there remain several directions for future exploration. First, multi-modal data fusion strategies, including depth maps and thermal infrared images, are planned to further enhance the model’s robustness under varying lighting conditions, occlusions, and complex backgrounds. Second, future work will focus on model compression and lightweight design to meet the real-time segmentation requirements of mobile or embedded devices, enabling efficient deployment. Finally, the model’s adaptability will be validated in greenhouse environments and under different climatic conditions to ensure stable performance across diverse production scenarios.

## Data Availability

The raw data supporting the conclusions of this article will be made available by the authors, without undue reservation.
